# *Transcription factor 7-like 2* rs77961654 polymorphism is related to stable angina and acute coronary syndrome in a Chinese population

**DOI:** 10.7150/ijms.108111

**Published:** 2025-03-31

**Authors:** Teng-Hung Yu, Wei-Hua Tang, Thung-Lip Lee, Chin-Feng Hsuan, Chia-Chang Hsu, Chao-Ping Wang, Ching-Ting Wei, Min-Chih Cheng, Fu-Mei Chung, Yau-Jiunn Lee, I-Ting Tsai

**Affiliations:** 1 Division of Cardiology, Department of Internal Medicine, E-Da Hospital, I-Shou University, Kaohsiung 82445, Taiwan.; 2 School of Medicine, College of Medicine, I-Shou University, Kaohsiung 82445, Taiwan.; 3 Division of Cardiology, Department of Internal Medicine, Taipei Veterans General Hospital, Yuli Branch, Hualien 98142, Taiwan.; 4 Faculty of Medicine, School of Medicine, National Yang Ming Chiao Tung University, Taipei 112304, Taiwan.; 5 School of Medicine for International Students, College of Medicine, I-Shou University, Kaohsiung 82445, Taiwan.; 6 Division of Cardiology, Department of Internal Medicine, E-Da Dachang Hospital, I-Shou University, Kaohsiung 807066, Taiwan.; 7 Division of Gastroenterology and Hepatology, Department of Internal Medicine, E-Da Hospital, I-Shou University, Kaohsiung 82445, Taiwan.; 8 Health Examination Center, E-Da Dachang Hospital, I-Shou University, Kaohsiung 807066, Taiwan.; 9 The School of Chinese Medicine for Post Baccalaureate, College of Medicine, I-Shou University, Kaohsiung 82445, Taiwan.; 10 Division of General Surgery, Department of Surgery, E-Da Hospital, I-Shou University, Kaohsiung 82445, Taiwan.; 11 Department of Psychiatry, Taipei Veterans General Hospital, Yuli Branch, Hualien 98142, Taiwan.; 12 Lee's Endocrinologic Clinic, Pingtung 90000, Taiwan.; 13 Department of Emergency, E-Da Hospital, I-Shou University, Kaohsiung 82445, Taiwan.

**Keywords:** acute coronary syndrome, polymorphism, stable angina, transcription factor 7-like 2

## Abstract

**Background:** Transcription factor 7-like 2 (TCF7L2) is a key member of the T-cell factor/lymphoid enhancer factor family, and it plays a pivotal role in human physiological processes and has been implicated in various pathological conditions. TCF7L2 has been associated with inflammation, metabolic regulation, and the development of atherosclerosis. Notably, TCF7L2 exerts an anti-atherosclerotic effect by activating specific molecular pathways. Furthermore, *TCF7L2* polymorphisms have been associated with the severity of coronary artery disease (CAD) and related mortality. This study aimed to investigate whether the *TCF7L2* gene polymorphism rs77961654 A/C influences the risk of CAD.

**Methods:** A total of 262 patients with acute coronary syndrome (ACS), 313 patients with stable angina, and 488 healthy individuals were enrolled. The rs77961654 variant of *TCF7L2* was genotyped.

**Results:** The frequency of the CC genotype of *TCF7L2* was higher in the stable angina and ACS groups compared to the healthy controls. After adjusting for confounding factors including age, sex, systolic blood pressure, body mass index, fasting blood glucose, total cholesterol, triglycerides, and smoking status, the CC genotype was associated with 10.46-fold and 8.00-fold higher risks of ACS and stable angina, respectively, than the AA genotype. In addition, the CC genotype was positively correlated with both stable angina and ACS, while the AA genotype was negatively correlated with ACS. Furthermore, the patients with stable angina or ACS carrying the CC genotype had significantly elevated levels of HbA1C, total white blood cell count, and lymphocyte count, and lower levels of adiponectin and secreted frizzled-related protein 5 compared to those with the AA genotype. A significant association was also identified between* TCF7L2* gene polymorphisms and type 2 diabetes mellitus (p for trend < 0.039).

**Conclusion:** Polymorphisms in the *TCF7L2* gene may be associated with a higher risk of stable angina and ACS.

## Introduction

Diabetes mellitus and coronary artery disease (CAD) share a complex and closely interlinked relationship, with diabetes being a major risk factor for cardiovascular complications 1. The pathogenesis of cardiovascular disease in individuals with diabetes is multifactorial, encompassing metabolic, inflammatory, and vascular disturbances. Chronic hyperglycemia, a hallmark of diabetes, promotes endothelial dysfunction and accelerates atherosclerosis through mechanisms such as oxidative stress, the formation of advanced glycation end-products, and persistent low-grade inflammation 2-4. In addition, common comorbidities in patients with diabetes such as dyslipidemia, insulin resistance, and hypertension further amplify the cardiovascular risk 5. The development of CAD in diabetic individuals is further complicated by an interplay between genetic predisposition and lifestyle factors, including diet and physical activity, which can either exacerbate or mitigate disease risk 6.

Genetic polymorphisms play a crucial role in the study of complex diseases, as they help elucidate biological pathways and identify molecular targets for novel therapies 7-9. In recent decades, numerous studies have identified that the *transcription factor 7-like 2 (TCF7L2)* gene is one of the strongest genetic determinants associated with susceptibility to type 2 diabetes mellitus (T2DM), particularly through the well-characterized rs7903146 polymorphism 10-12. This variant has been shown to influence insulin secretion and glucose homeostasis, increasing vulnerability to T2DM 13. The impact of this genetic variant on the Wnt signaling pathway, essential for pancreatic beta cell function and insulin production, underscores its role in the pathophysiology of T2DM 13,14. Impaired insulin secretion caused by *TCF7L2* polymorphisms contributes to chronic hyperglycemia, which is a significant driver of cardiovascular complications. Consequently, individuals carrying *TCF7L2* risk variants are not only predisposed to T2DM but also have an elevated risk of CAD.

A nonsynonymous single nucleotide variant has been identified in the exonic region of the *TCF7L2* gene on chromosome 10, where the reference allele is C and the observed variant is A. An affected individual is heterozygous, meaning that only one of the two chromosomes carries this mutation. The dbSNP ID of this variant is rs77961654. According to bioinformatics predictions, the SIFT score is 0.15, suggesting a relatively low impact on protein function, while the PolyPhen2 score is 0.996, indicating that this mutation is highly likely to have a damaging effect on protein structure or function. However, this variant remains relatively understudied 15,16. Although emerging evidence suggests that rs77961654 may influence blood glucose regulation by affecting insulin secretion and function 15,16, no previous studies have reported an association between this polymorphism and CAD. We designed this study to explore whether the *TCF7L2* gene variant rs77961654 A/C affects susceptibility to stable angina and acute coronary syndrome (ACS), and also the associations with clinical and biochemical characteristics in Chinese patients residing in Taiwan.

## Methods

### Participants

This study was conducted from December 2023 to October 2024 at the Cardiovascular Ward of Kaohsiung E-Da Hospital, Taiwan, and included patients with ACS (n=262) and stable angina (n=313). Significant CAD was diagnosed in patients presenting with CAD-related symptoms and ≥50% diameter stenosis in one or more coronary arteries. The CAD patients were subdivided into two groups: (1) the ACS group, including patients with unstable angina pectoris, non-ST-segment elevation myocardial infarction, or ST-segment elevation myocardial infarction, and (2) the stable angina group, comprising patients with stable angina pectoris. The exclusion criteria were patients who had heart failure, renal dysfunction, hepatic dysfunction, or valvular heart disease. The control group (n=488) were individuals who underwent health examinations at Kaohsiung E-Da Hospital. The inclusion criteria were participants who were unrelated with no identifiable cardiovascular risk factors, no prior history of CAD, and not currently on any medications. The exclusion criteria were any history or signs of cardiovascular disease, as well as the presence of any significant comorbidities such as heart failure, hyperlipidemia, renal dysfunction, hepatic dysfunction, valvular heart disease, hypertension, or T2DM. The health status of all participants was confirmed through routine medical examinations and self-reported medical histories to ensure compliance with the eligibility criteria. The study protocol was approved by the Human Research Ethics Committee of E-Da Hospital, and informed consent was obtained from all participants prior to enrollment.

### Data collection

All of the enrolled participants were ethnically Han Chinese and resided in southern Taiwan. Information regarding personal medical history, tobacco and alcohol consumption was collected through standardized questionnaires. Tobacco and alcohol consumption were categorized as current, former (abstained for ≥1 year), or never. Those classified in the former and current categories were combined into a single group for analysis 17. Body mass index (BMI) and waist circumference (measured midway between the lowest rib and the top of the hip) and hip circumference (measured at the widest part of the hips) were also recorded, and waist-to-hip ratio (WHR) was calculated using the mean of two measurements. Systolic/diastolic blood pressure (SBP/DBP) was measured following standard protocols with an electronic sphygmomanometer (HEM-907, Omron, Japan). The modified Simpson's method with four-chamber apical view was used to calculate left ventricular ejection fraction 18. Hyperlipidemia was diagnosed according to the ATP III criteria if any of the following were present: (1) total cholesterol ≥200 mg/dL, (2) low-density lipoprotein cholesterol (LDL-C) ≥130 mg/dL, (3) triglycerides ≥150 mg/dL, (4) high-density lipoprotein cholesterol (HDL-C) <35 mg/dL in men or <39 mg/dL in women, or (5) currently receiving lipid-lowering medications 19. Hypertension was defined as having prescriptions for antihypertensive medication, or SBP/DBP ≥140/90 mmHg. T2DM was defined as a level of fasting glucose >126 mg/dL, or receiving antidiabetic medications 20.

### Laboratory measurements

All laboratory measurements were made using blood samples obtained after an overnight fast, and included serum triglycerides, complete blood cell count, glucose, HDL-C, uric acid, LDL-C, total cholesterol, blood urea nitrogen (BUN), and albumin 21. Levels of hemoglobin A1c were evaluated with an automatic analyzer (HLC-723G8, Tosoh Corp., Tokyo, Japan). Serum creatinine levels were determined using the Jaffe method. Chemiluminescent microparticle immunoassays were used to assess troponin I and CK-MB levels in serum. Total leukocyte count and proportions of lymphocytes, neutrophils, and monocytes were measured using a hematology analyzer (XE-2100, Sysmex, Kobe, Japan). Absolute counts for each leukocyte subtype were measured by multiplying its proportion by the total leukocyte count. Estimated glomerular filtration rate (eGFR) was determined using the CKD-EPI formula 22.

### Plasma secreted frizzled-related protein 5 (Sfrp5), adiponectin, and high-sensitivity C-reactive protein (hs-CRP) measurements

Plasma from blood samples obtained after an overnight fast was kept at -80°C before analysis. Enzyme-linked immunosorbent assay (ELISA) kits were used to measure concentrations of Sfrp5 following the manufacturers' instructions (Cloud- Clone Corp., Katy, USA). The intra- and inter-assay coefficients of variation (COV) of the assay were <10% (n=3) and <12% (n=3), respectively. Concentrations of hs-CRP in the plasma samples were measured using an immunochemistry device (Beckman Coulter IMMAGE, Brea, CA), with each assay being performed twice in one experiment, and a detection limit of 0.2 mg/L. Another ELISA kit (B-Bridge International, Sunnyvale, CA) was used to measure plasma concentrations of adiponectin, with sample dilution curves run in parallel to the standard curve. The inter-assay COV was 3.2-7.3% (n=3), and the intra-assay COV was 3.1-6.2% (n=4).

### Determining the *TCF7L2* genotype (rs77961654)

A DNA Blood Mini Kit (QIAamp, Qiagen, Valencia, CA) was used to extract genomic DNA from whole blood samples. Extracted DNA was dissolved in Tris/EDTA buffer [10 mM Tris, 1 mM EDTA (pH 7.8)], quantified by OD260 measurement, and refrigerated at -20°C before being used as a template for PCR. SNP rs77961654 in the *TCF7L2* gene was genotyped using a QuantStudio 3 real-time PCR system with a TaqMan^TM^ SNP Genotyping Assay (Thermo Fisher Scientific). Genotyping was performed using the predesigned TaqMan assay for rs77961654 (C-104679642-10). The context sequence for rs77961654 (C-104679642-10) is AGGTGAAGGC AGCTGCCTCAGCCCA**[A/C]**CCTCTTCAGATGGAAGCTTACTAGA. In brief, genomic DNA (5 ng) was amplified in a final volume of 10 μL which contained 40× Predesigned TaqManTM SNP Genotyping assay (0.25 ml; comprised of two unlabeled primers, VIC® and FAM™ dye-labeled MGB probes) and 2× TaqMan® GTXpress™ Master Mix (5 ml). The PCR conditions were initial denaturation at 95°C for 2 min, followed by 40 cycles at 95°C for 15 sec and 60°C for 40 sec. Genotyping analysis was performed with allelic discrimination plots with traces using real-time PCR data.

### Statistical analysis

Normally distributed continuous data are shown in mean (SD), and non-normally distributed data in median (interquartile range). The Kolmogorov-Smirnov test was used to assess the normality of data. Due to skewed distributions, plasma concentrations of troponin I, CK-MB, BUN, triglycerides, Sfrp5, adiponectin, and hs-CRP were logarithmically transformed prior to being analyzed. Unpaired Student's *t*-tests and *chi*-square tests were applied, as appropriate, for comparisons among the stable angina, ACS and control groups. A Pearson's correlation heatmap was generated to assess associations between *TCF7L2* genotypes with the incidence of stable angina and ACS. Associations between stable angina and ACS with the frequency of genotypes were evaluated with logistic regression analyses, adjusting for sex, SBP, triglycerides, total cholesterol, fasting glucose, smoking status, BMI, and age. Results are presented as odds ratio (OR) with 95% confidence interval (CI). Continuous variables across the three *TCF7L2* genotype groups were compared with one-way analysis of variance. The prevalence of T2DM and trends across the *TCF7L2* genotype groups were assessed with the *chi*-square test and Cochran-Armitage trend test, respectively. Statistical analysis was performed with JMP v7.0 for Windows (SAS Institute, Cary, NC). P values < 0.05 were considered statistically significant.

## Results

### Characteristics of the three study groups

Both the ACS and stable angina groups were significantly older, had higher rates of male patients and current tobacco smokers/alcohol drinkers, along with higher SBP, BMI, waist circumference and WHR compared to the controls (Table 1). In addition, compared to the stable angina group, the ACS group had a significantly higher prevalence of left anterior descending artery stenosis and two-vessel disease, and lower prevalence of single-vessel disease.

### Biochemical characteristics of the three study groups

The ACS group had significantly elevated levels of fasting blood glucose, HbA1C, triglycerides, uric acid, BUN, creatinine, total white blood cell (WBC) count, lymphocytes, monocytes, neutrophils, and hs-CRP, along with lower eGFR, total cholesterol, LDL-C, HDL-C, hemoglobin, and albumin than the controls (Table 2). In addition, compared to the control group, the stable angina group had significantly elevated levels of BUN, fasting blood glucose, creatinine, HbA1C, triglycerides, uric acid, total WBC count, neutrophils, monocytes, lymphocytes, and hs-CRP, along with lower levels of total cholesterol, eGFR, albumin, LDL-C, hemoglobin and HDL-C. Moreover, the ACS group had significantly higher levels of LDL-C, total cholesterol, total WBC count, neutrophils, monocytes, lymphocytes, creatine kinase-MB, troponin I, and fasting blood glucose, along with lower levels of albumin, eGFR, and HDL-C compared to the stable angina group.

### Associations between *TCF7L2* genotypes with stable angina and ACS

**Figure 1:**
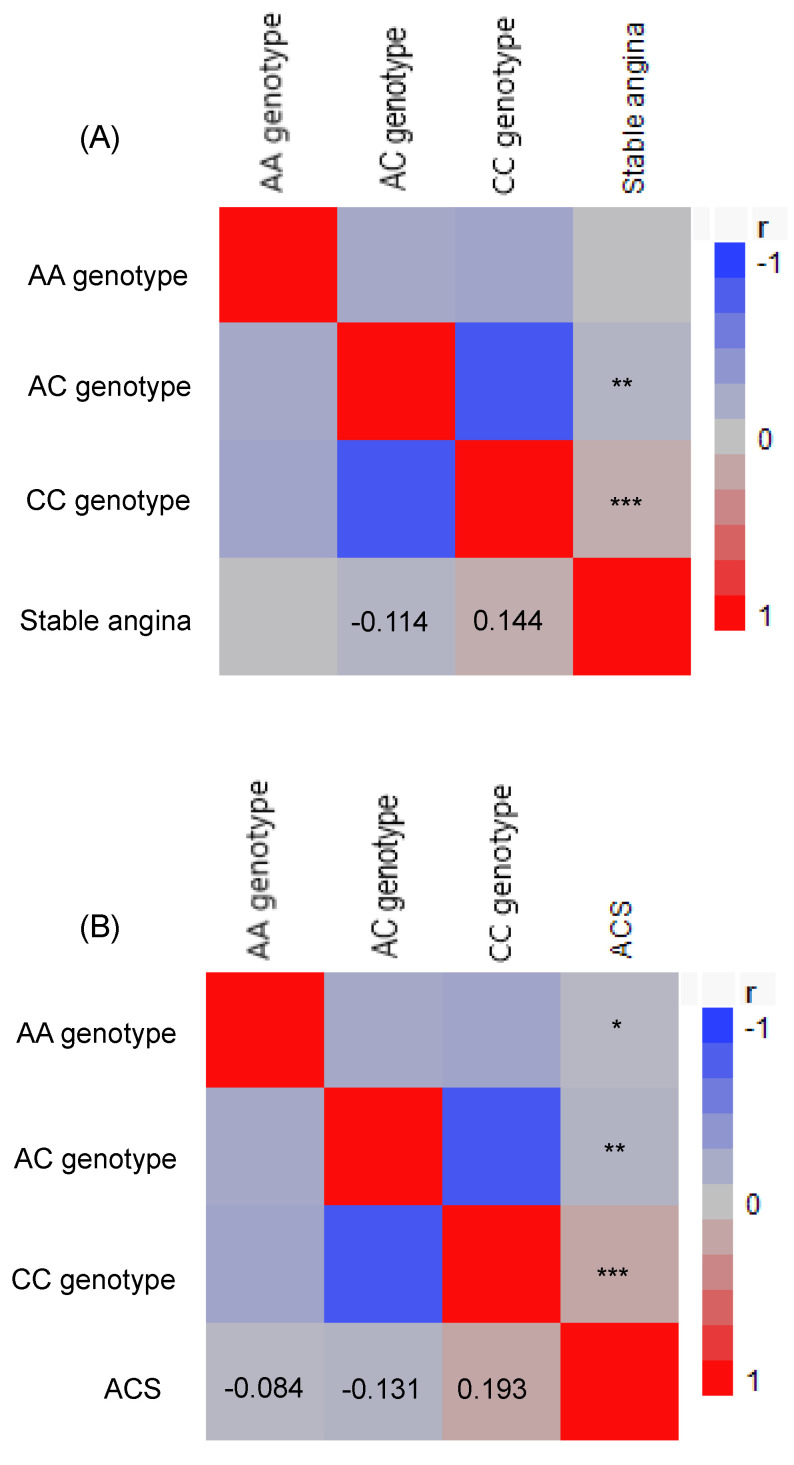
A Pearson correlation heat map was generated to explore the relationship between the genotypes of the *transcription factor 7-like 2* and the occurrence of stable angina (A) and acute coronary syndrome (B) among patients. The red color indicates a positive correlation, whereas the blue and grey colors indicate a negative correlation. *p <0.05, **p <0.001, and ***p <0.0001. ACS, acute coronary syndrome.

*TCF7L2* gene A/C genotype distributions in the three study groups were in Hardy-Weinberg equilibrium (Table 3). There was a significant difference in *TCF7L2* genotype distribution between the stable angina/ACS groups and control group (p < 0.0001). A higher prevalence of the CC genotype was observed in both the stable angina and ACS groups, suggesting an association between *TCF7L2* gene polymorphisms and the presence of stable angina and ACS. Specifically, the CC genotype was associated with increased odds of stable angina (CC vs. AA: crude OR = 1.93, 95% CI = 1.15-3.34, p = 0.013) and ACS (OR = 4.09, 95% CI = 2.09-8.77, p < 0.0001). The associations between the *TCF7L2 CC* genotype and both stable angina and ACS remained significant after adjustments in logistic regression for triglycerides, age, sex, SBP, BMI, fasting glucose, total cholesterol, and smoking status. The adjusted OR for stable angina was 8.00 (95% CI = 3.09-21.96, p < 0.0001), and for ACS, it was 10.46 (95% CI = 3.56-13.94, p < 0.0001) when comparing the CC genotype to the AA genotype. As shown in Table 3, the CC genotype was associated with significantly elevated risks of stable angina and ACS compared to the AA/AC genotypes. Moreover, Pearson's correlation analysis revealed that the *TCF7L2* CC genotype was positively correlated with both stable angina (r = 0.144, p < 0.0001) and ACS (r = 0.193, p < 0.0001). Conversely, the *TCF7L2* AA genotype was negatively correlated with ACS (r = -0.084, p = 0.022), and the *TCF7L2* AC genotype was negatively correlated with both stable angina (r = -0.114, p = 0.001) and ACS (r = -0.131, p = 0.0003) (Figure 1).

### Biochemical and clinical characteristics of the patients with CAD according to *TCF7L2* genotype

**Figure 2:**
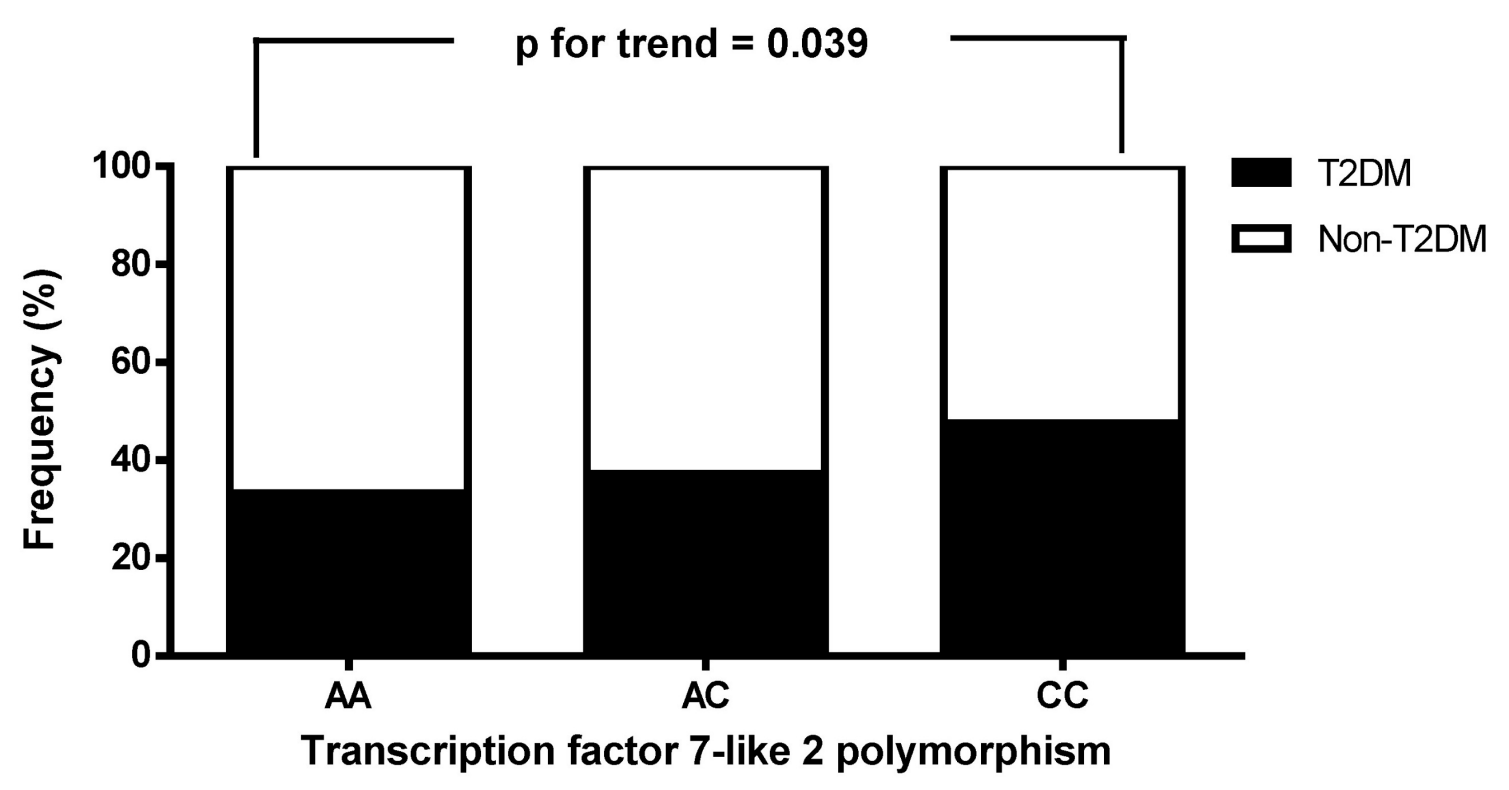
The distribution of patients with stable angina and acute coronary syndrome, stratified by the presence or absence of type 2 diabetes mellitus (T2DM), was analyzed according to the genotypes of *transcription factor 7-like 2* (*TCF7L2*). A significant association was observed between T2DM status and *TCF7L2* genotypes.

The biochemical and clinical characteristics of the 575 CAD patients stratified by *TCF7L2* genotypes are shown in Table 4. The prevalence rates of the AA, AC, and CC genotypes were 5.7%, 35.5%, and 58.8%, respectively. Patients with the CC genotype had higher levels of HbA1C, total WBC count, and lymphocyte count, along with lower levels of adiponectin and Sfrp5, compared to those with the AA genotype. No significant differences were observed among the three *TCF7L2* genotypes with respect to hs-CRP, BMI, eGFR, waist circumference, triglycerides, total cholesterol, DBP, fasting glucose, creatinine, hemoglobin, LDL-C, uric acid, monocyte count, albumin, BUN, sex, SBP, WHR, neutrophil count, or HDL-C. We also analyzed the frequency of T2DM status (absence or presence of T2DM) in the patients with stable angina and ACS, stratified by *TCF7L2* genotypes. T2DM status was found to be significantly associated with *TCF7L2* genotypes (absence vs. presence of T2DM: AA/AC/CC = 66.7%/62.8%/52.4% vs. 33.3%/37.2%/47.6%, p for trend = 0.039; Figure 2).

## Discussion

This study examined the relationship between the *TCF7L2* gene variant rs77961654 A/C and the risk of stable angina and ACS. There were three key findings: (1) the *TCF7L2* CC genotype was more prevalent in the patients with stable angina and ACS than in the controls; (2) in both univariate and multivariate logistic regression analyses adjusted for age, sex, SBP, BMI, fasting glucose, total cholesterol, triglycerides, and smoking status, the *TCF7L2* CC genotype was independently associated with an increased risk of stable angina and ACS; (3) the patients with ACS and stable angina who were CC genotype carriers had significantly higher HbA1C, total WBC count, and lymphocyte count, along with lower adiponectin and Sfrp5 levels compared to AA genotype carriers. Furthermore, significant associations were also observed between *TCF7L2* gene polymorphisms and T2DM. To our knowledge, this is the first study to report an association between the *TCF7L2* rs77961654 polymorphism and both stable angina and ACS.

T2DM and CAD are closely related, with T2DM significantly increasing the risk of CAD 23,24. The metabolic abnormalities associated with T2DM induce oxidative stress and inflammation and consequently accelerate atherosclerosis in coronary arteries, which is central to the pathogenesis of CAD 2,3,25. Chronic hyperglycemia damages the arterial endothelium, impairing the nitric oxide production essential for vascular relaxation. This then promotes endothelial dysfunction, reducing vascular elasticity and contributing to hypertension, which further predisposes diabetic patients to CAD 26,27. T2DM has also been associated with a pro-inflammatory and pro-thrombotic state, with elevated levels of cytokines and inflammatory markers that drive vascular inflammation and thrombogenesis 1,28,29. These mechanisms may explain why CAD is the leading cause of mortality in individuals with T2DM.

The first key finding of this study is that the* TCF7L2* CC genotype was more prevalent in the patients with stable angina and ACS than in the controls. Previous studies have reported a significant association between rs12255372 in the *TCF7L2* gene with susceptibility to T2DM in the global population 30,31. Genetic polymorphisms significantly contribute to the risk of T2DM by influencing glucose metabolism, insulin sensitivity, and beta cell function 15,32-34. Variants in genes such as *TCF7L2*, *PPAR-γ*, and *KCNJ11* have been strongly associated with an increased risk of T2DM, with each playing a distinct role in the pathophysiology of the disease 35-37. Importantly, various diabetes-related polymorphisms have also been linked with CAD, highlighting the genetic overlap and shared pathophysiological mechanisms between the two conditions. For example, the* TCF7L2* variant rs7903146 has been shown to be a significant genetic predictor of T2DM and to be associated with an increased risk of CAD 38-41. In addition, PPAR-γ polymorphisms such as Pro12Ala have been shown to affect insulin sensitivity and lipid metabolism, thereby contributing to both T2DM and CAD through effects on adipocyte differentiation, inflammation, and endothelial function 36. Moreover, the KCNJ11 E23K variant has been associated with T2DM due to its impact on beta cell function and insulin release, and it has also been linked to cardiac arrhythmias in diabetic patients 37. These findings highlight the genetic link between T2DM and CAD, with certain polymorphisms affecting both metabolic and cardiovascular health and increasing the risk of both conditions.

The second finding of this study is that the *TCF21* CC homozygote genotype was associated with both stable angina and ACS in the enrolled Han Taiwanese patients. The *TCF7L2* gene is a key genetic marker for T2DM susceptibility, with associations identified in a genome-wide association study 42. Located on chromosome 10q25.2, *TCF7L2* influences glucose metabolism by regulating insulin secretion, beta cell function, and insulin sensitivity via the Wnt signaling pathway, which affects pancreatic beta cells. Polymorphisms such as rs7903146 disrupt this pathway and consequently impair insulin production, a major factor in the pathogenesis of T2DM. Carriers of the T allele of rs7903146 have been shown to have increased risks of impaired glucose tolerance and reduced insulinotropic response to GLP-1, leading to insulin resistance, dyslipidemia, and a pro-inflammatory state, which are also risk factors for CAD 39,43- 45. Thus, high-risk *TCF7L2* variants are linked to a higher prevalence of CAD in T2DM patients, highlighting the dual role of *TCF7L2* in metabolic and cardiovascular health. In addition to rs7903146, other *TCF7L2* polymorphisms such as rs12255372, rs4506565, rs11196205, and rs11196218 have been associated with diabetes, glucose homeostasis, and cardiovascular conditions 30,31,46-48. In this study we investigated the rs77961654 polymorphism, a comparatively understudied polymorphism of *TCF7L2* which affects gene exons in patients with CAD 15,16. Notably, we found that rs77961654 was associated with HbA1c levels, and that the CC genotype was significantly associated with both stable angina and ACS (see Table 3 and Figure 1).

The third key finding of this study is that the CC genotype carriers with both stable angina and ACS had significantly higher HbA1C, total WBC count, and lymphocyte count compared to those with the AA genotype, along with lower adiponectin and Sfrp5 levels (Table 4). In addition, the *TCF7L2* genotypes were significantly associated with T2DM (Figure 2). Our findings may indicate a greater susceptibility to CAD among individuals with the CC genotype, although the precise molecular mechanisms remain unknown. Adiponectin and Sfrp5 are anti-inflammatory adipokines with protective roles in metabolic and inflammatory processes which are relevant to the pathophysiology of CAD 49-51. Adiponectin reduces monocyte adhesion, foam cell formation, and systemic inflammation by enhancing insulin sensitivity and inhibiting pro-inflammatory cytokines such as IL-6 and TNF-α 52-54. Similarly, Sfrp5 modulates Wnt signaling to reduce inflammation in adipose tissue, thereby limiting vascular damage 55,56. Low adiponectin and Sfrp5 levels have been associated with increased inflammation and vascular injury, which in turn are associated with the development and progression of CAD 55-57. TCF7L2 is a downstream transcription factor in the canonical Wnt/β-catenin pathway, and it has been shown to be involved in insulin synthesis, vascular cell proliferation, inflammation, and plaque formation, all of which are key processes in atherosclerosis 39,58. Conversely, Sfrp5 antagonizes Wnt signaling, particularly the non-canonical pathway, by binding to Wnt ligands, thereby reducing inflammation and oxidative stress in adipose and vascular tissues 59,60, which may protect against metabolic syndrome and CAD 61,62. While TCF7L2 and Sfrp5 do not interact directly, they play complementary or opposing roles in Wnt signaling 13,55, potentially influencing metabolic and inflammatory pathways related to CAD. In the present study, low levels of Sfrp5 may have exacerbated conditions influenced by *TCF7L2* polymorphisms or mutations, such as impaired insulin secretion and glucose metabolism. Thus, *TCF7L2* polymorphisms and Sfrp5 may modulate the effect of each other on metabolic and inflammatory processes relevant to CAD. Similarly, while there is no direct evidence of an interaction between TCF7L2 and adiponectin, alterations in TCF7L2 that contribute to insulin resistance could influence adiponectin levels or sensitivity 63. Individuals with *TCF7L2* polymorphisms associated with a predisposition to T2DM often have metabolic profiles characterized by low adiponectin levels 63. In addition, inflammation is a primary pathological mechanism underlying CAD 64. In this study, we found that the patients with the *TCF7L2* rs77961654 CC genotype had reduced levels of adiponectin and Sfrp5, along with elevated HbA1c, total WBC count, and lymphocyte count, all of which are associated with an increased risk of CAD. Further research is required to clarify the underlying mechanisms and molecular interactions contributing to these associations.

The demographic characteristics of the patients with CAD in our study are consistent with other studies on CAD, including being significantly older, predominantly male, and having higher rates of current smoking, alcohol consumption, BMI, waist circumference, WHR, and SBP compared to the control group (Table 1) 65. As expected, the ACS subgroup had a significantly higher prevalence of two-vessel disease and left anterior descending artery stenosis, along with a lower incidence of single-vessel disease compared to the stable angina subgroup (Table 1).

Several limitations of this study should be acknowledged. First, the relatively small number of participants. In addition, we could not infer causality between *TCF7L2* rs77961654 A/C variants with the risk of stable angina and ACS due to the cross-sectional design of the study. Longitudinal studies are warranted to confirm the role of *TCF7L2* gene polymorphisms in relation to the risk of stable angina and ACS. Furthermore, validation of the results is needed in other ethnic groups or independent cohorts. Prospective studies are also recommended to verify our results.

## Conclusions

The *TCF7L2* gene plays a crucial role in both the onset and progression of T2DM through its involvement in insulin secretion and glucose homeostasis, while its association with CAD underscores its broader influence on metabolic and vascular health. Our findings suggest that the *TCF7L2* rs77961654 polymorphism may be associated with CAD, characterized by distinct levels of HbA1C, total WBC count, lymphocyte count, adiponectin, and Sfrp5. These findings have important clinical implications. First, the* TCF7L2* rs77961654 polymorphism may contribute to the development of CAD by influencing key metabolic and inflammatory markers. The observed associations with HbA1c, total WBC count, lymphocyte count, adiponectin, and Sfrp5 suggest potential links between this genetic variant and glycemic control, immune response, and adipokine signaling, all of which are critical in the pathophysiology of CAD. Second, identifying individuals carrying this polymorphism could aid in risk stratification and personalized management of CAD, particularly in patients with coexisting metabolic or inflammatory conditions. Future research may explore whether targeted therapeutic interventions such as glucose-lowering strategies, anti-inflammatory treatments, or adipokine modulation can mitigate the risk of CAD in these individuals. Moreover, integrating genetic screening into routine cardiovascular risk assessment may enhance early detection and prevention strategies. While the exact mechanisms remain to be elucidated, continued research and the development of therapies targeting the *TCF7L2* rs77961654 polymorphism could improve diabetes and cardiovascular health outcomes in genetically susceptible individuals.

## Figures and Tables

**Table 1:** Distributions of demographic characteristics in 488 controls and 575 stable angina or ACS patients

Parameters	Controls	ACS	Stable angina	p-value^a^	p-value^b^	p-value^c^
N	488	262	313			
Age (years)	48.2±12.6	64.4±12.8	62.6±11.1	**<0.0001**	**<0.0001**	0.083
Sex (male/female)	257/231	215/47	241/72	**<0.0001**	**<0.0001**	0.136
Hypertension (n, %)	-	171(65.3)	215(68.7)	-	-	0.384
Type 2 diabetes mellitus (n, %)	-	104(39.7)	130(41.5)	-	-	0.655
Hyperlipidemia (n, %)	-	183(69.9)	211(67.4)	-	-	0.531
Current smoker (n, %)	93(19.1)	116(44.3)	130(41.5)	**<0.0001**	**<0.0001**	0.503
Drinking (n, %)	34(7.0)	78(29.8)	116(37.1)	**<0.0001**	**<0.0001**	0.086
Body mass index (kg/m^2^)	24.3±4.2	26.3±4.0	26.4±3.8	**<0.0001**	**<0.0001**	0.670
Waist circumference (cm)	81.4±11.8	92.5±10.2	92.5±9.6	**<0.0001**	**<0.0001**	0.994
WHR	0.85±0.08	0.95±0.08	0.94±0.07	**<0.0001**	**<0.0001**	0.057
Systolic blood pressure (mmHg)	125±18	133±23	133±19	**<0.0001**	**<0.0001**	0.746
Diastolic blood pressure (mmHg)	77±12	76±15	78±12	0.373	0.702	0.280
LVMI	-	124.8±35.6	121.1±35.7	-	-	0.299
LVEF (%)	-	60.6±11.3	62.3±12.2	-	-	0.114
Number of diseased vessels (%)						
Single-vessel disease	-	98(37.4)	143(45.7)	-	-	**0.045**
Two-vessel disease	-	83(31.7)	76(24.3)	-	-	**0.048**
Three-vessel disease	-	81(30.9)	94(30.0)	-	-	0.819
Location of stenosis				-	-	
Left anterior descending	-	217(82.8)	216(69.0)	-	-	**0.0001**
Left circumflex	-	135(51.5)	178(56.9)	-	-	0.200
Right coronary artery	-	157(59.9)	182(58.2)	-	-	0.666
Gensini score	-	35.0(12.5-64.0)	24.5(8.0-61.3)	-	-	0.304

Data are expressed as mean ± SD, number (percentage), or median (interquartile range). WHR, waist-to-hip ratio; ACS, acute coronary syndrome; LVMI, left ventricular mass index; LVEF, left ventricular ejection fraction; ^a^: Data were compared between ACS patients and controls. ^b^: Data were compared between stable angina patients and controls. ^c^: Data were compared between ACS patients and stable angina patients.

**Table 2:** Biochemical characteristics in 488 controls and 575 stable angina or acute coronary syndrome patients

Parameters	Controls	ACS	Stable angina	p-value^a^	p-value^b^	p-value^c^
No.	488	262	313			
Fasting sugar (mg/dL)	94.9±16.0	156.7±78.6	131.7±62.3	**<0.0001**	**<0.0001**	**<0.0001**
HbA1C (%)	5.6±0.7	7.0±1.8	6.9±1.7	**<0.0001**	**<0.0001**	0.653
Total cholesterol (mg/dL)	205.4±40.4	181.3±44.6	172.3±44.0	**<0.0001**	**<0.0001**	**0.015**
Triglyceride (mg/dL)	95.0(64.0-144.8)	126.0(91.8-199.3)	117.0(87.0-170.0)	**<0.0001**	**<0.0001**	0.097
HDL-cholesterol (mg/dL)	59.3±18.1	38.8±11.7	41.9±11.7	**<0.0001**	**<0.0001**	**0.002**
LDL-cholesterol (mg/dL)	129.1±37.3	109.9±38.9	100.9±37.1	**<0.0001**	**<0.0001**	**0.005**
Uric acid (mg/dL)	5.9±1.4	6.8±2.0	6.5±2.3	**<0.0001**	**<0.0001**	0.187
BUN (mg/dl)	10.7(8.7-13.1)	17.6(13.8-23.0)	18.0(13.5-22.4)	**<0.0001**	**<0.0001**	0.797
Creatinine (mg/dl)	0.8±0.2	1.5±1.2	1.4±1.1	**<0.0001**	**<0.0001**	0.140
eGFR (ml/min/1.73m^2^)	128.4±36.2	64.9±28.1	70.2±28.5	**<0.0001**	**<0.0001**	**0.025**
Hemoglobin (g/dl)	14.3±1.7	13.9±2.2	13.6±2.0	**0.006**	**<0.0001**	0.055
Albumin (g/L)	4.6±0.3	3.9±0.4	4.0±0.4	**<0.0001**	**<0.0001**	**0.001**
Total WBC count (10^9^/L)	5.804±1.601	9.519±3.824	7.631±2.903	**<0.0001**	**<0.0001**	**<0.0001**
Neutrophil count (10^9^/L)	3452±1232	6455±3356	4866±2513	**<0.0001**	**0.0001**	**<0.0001**
Monocyte count (10^9^/L)	336±142	549±307	450±214	**<0.0001**	**<0.0001**	**<0.0001**
Lymphocyte count (10^9^/L)	1830±566	2254±1281	2056±847	**<0.0001**	**<0.0001**	**0.032**
Creatine kinase-MB (ng/mL)	-	4.9(1.7-22.4)	2.1(1.1-7.1)	-	-	**<0.0001**
Troponin I (ng/mL)	-	0.4(0.1-2.6)	0.1(0.0-0.8)	-	-	**<0.0001**
hs-CRP (mg/L)	0.8(0.4-1.9)	3.2(0.7-7.7)	2.2(0.8-6.2)	**<0.0001**	**<0.0001**	0.664

Data are expressed as mean ± SD, or median (interquartile range). ACS, acute coronary syndrome; HDL, high-density lipoprotein; LDL, low-density lipoprotein; BUN, blood urine nitrogen; eGFR, estimated glomerular filtration rate; WBC, white blood cell; hs-CRP, high sensitivity C-reactive protein.^ a^: Data were compared between ACS patients and controls. ^b^: Data were compared between stable angina patients and controls. ^c^: Data were compared between ACS patients and stable angina patients.

**Table 3:** Adjusted odds ratio and 95% confidence interval of stable angina and acute coronary syndrome with *TCF7L2* genotypic frequencies

Parameter	Controls (n=488)	Stable angina (n=313)	Crude OR	95% CI	AOR^a^	95% CI	p-value
***TCF7L2* genotypes**							
AA genotype^b^	41(8.4)	23 (7.4)	1.00	-	1.00	-	-
AC genotype	241(49.4)	113 (36.1)	1.05	0.62-1.83	2.56	1.02-6.70	**0.045**
CC genotype	206(42.2)	177 (56.5)	1.93	1.15-3.34	8.00	3.09-21.96	**<0.0001**
AA/AC genotype^b^	282 (57.8)	136 (43.5)	1.00	-	1.00	-	-
CC genotype	206 (42.2)	177 (56.5)	1.85	1.39-2.47	3.60	2.11-6.28	**<0.0001**
	Controls (n=488)	ACS (n=262)	Crude OR	95% CI	AOR^a^	95% CI	p-value
***TCF7L2* genotypes**							
AA genotype^b^	41(8.4)	10(3.8)	1.00	-	1.00	-	-
AC genotype	241(49.4)	91(34.7)	1.97	1.00-4.26	2.25	0.77-7.10	0.141
CC genotype	206(42.2)	161(61.5)	4.09	2.09-8.77	10.46	3.56-13.94	**<0.0001**
AA/AC genotype^b^	282 (57.8)	101 (38.6)	1.00	-	1.00	-	-
CC genotype	206 (42.2)	161(61.5)	2.28	1.68-3.10	5.34	2.94-10.04	**<0.0001**

ACS, acute coronary syndrome; TCF7L2, transcription factor 7-like 2. AOR, adjusted odds ratio; CI, confidence interval. ^a^: The adjusted ORs with their 95% CI were estimated by employing multiple logistic regression models, after controlling for age, sex, systolic blood pressure, body mass index, fasting sugar, total cholesterol, triglyceride, and smoking. ^b^: as a reference group.

**Table 4:** Clinical and biochemical parameters of the studied patients with CAD in different genotypes of transcription factor 7-like 2

Parameters	AA	AC	CC	p-value
No.	33	204	338	
Age	66.5±10.6	62.0±10.6	64.0±12.7	**0.047**
Sex (male/female)	28/5	158/46	270/68	0.522
Body mass index (kg/m^2^)	26.1±4.1	26.1±3.9	26.5±3.9	0.548
Waist circumference (cm)	92.3±9.8	92.0±10.8	92.9±9.3	0.647
WHR	0.95±0.06	0.94±0.08	0.94±0.07	0.617
Systolic blood pressure (mmHg)	137±19	132±20	134±21	0.423
Diastolic blood pressure (mmHg)	81±16	77±13	77±14	0.221
Fasting sugar (mg/dL)	138.4±56.1	143.8±72.2	143.1±72.1	0.922
HbA1C (%)	6.7±1.5	6.8±1.6	7.2±1.9	**0.034**
Total-cholesterol (mg/dL)	175.9±45.4	171.9±42.7	179.2±45.4	0.178
Triglyceride (mg/dL)	114.0(78.0-152.0)	123.0(88.0-179.5)	120.0(90.0-184.0)	0.580
HDL-cholesterol (mg/dL)	39.6±11.6	41.0±11.2	40.3±12.2	0.743
LDL-cholesterol (mg/dL)	103.6±38.4	100.7±35.3	107.7±39.7	0.112
Uric acid (mg/dL)	6.3±2.1	5.9±1.6	6.1±1.8	0.209
BUN (mg/dl)	15.6(13.8-20.9)	18.0(13.5-23.4)	17.7(13.7-22.5)	0.851
Creatinine (mg/dl)	1.1(1.0-1.3)	1.2(1.0-1.4)	1.2(1.0-1.4)	0.538
eGFR (ml/min/1.73m^2^)	68.0±21.7	68.7±30.4	67.2±27.7	0.828
Hemoglobin (g/dl)	13.6±2.0	13.5±2.2	13.9±2.1	0.146
Albumin (g/L)	3.9±0.5	4.0±0.4	4.0±0.4	0.751
Total WBC count (10^9^/L)	7.812±2.601	8.093±3.117	8.806±3.733	**0.035**
Neutrophil count (10^9^/L)	5391±2484	5293±2727	5812±3251	0.156
Monocyte count (10^9^/L)	453±211	474±225	513±292	0.166
Lymphocyte count (10^9^/L)	1752±716	2094±994	2221±1139	**0.039**
hs-CRP (mg/L)	2.6(0.7-6.9)	2.3(0.8-7.0)	3.5(0.4-11.9)	0.732
Adiponectin (μg/mL)	5.1(3.3-12.0)	3.5(2.1-7.1)	2.5(1.4-4.8)	**0.005**
Sfrp5 (ng/mL)	8.5(4.8-14.2)	6.8(4.3-10.1)	6.3(4.0-10.5)	**0.035**

Data are expressed as mean ± SD, or median (interquartile range). WHR, waist-hip ratio; HDL, high-density lipoprotein; LDL, low-density lipoprotein; BUN, blood urine nitrogen; eGFR, estimated glomerular filtration rate; WBC, white blood cell; hs-CRP, high sensitivity C-reactive protein; Sfrp, secreted frizzled-related protein.
